# Supplementary figures and images for: Depleting Cationic Lipids Involved in Antimicrobial Resistance Drives Adaptive Lipid Remodeling in Enterococcus faecalis
Source: mBio. 2023 Jan 11;14(1):e03073-22. doi: 10.1128/mbio.03073-22 (PMC9973042; doi:10.1128/mbio.03073-22)

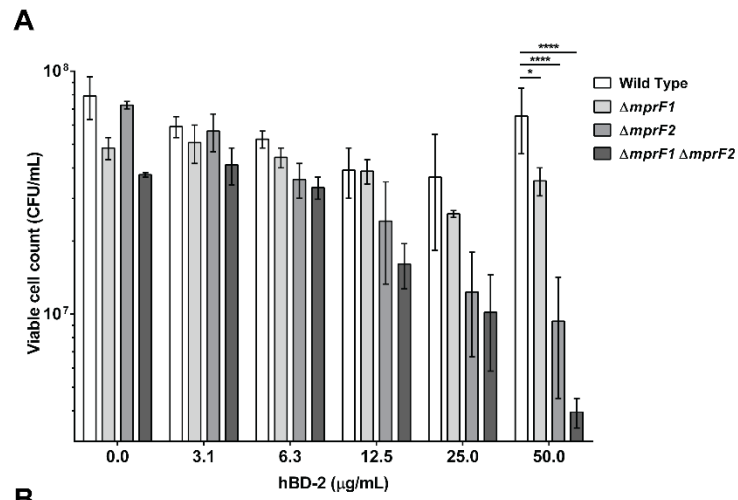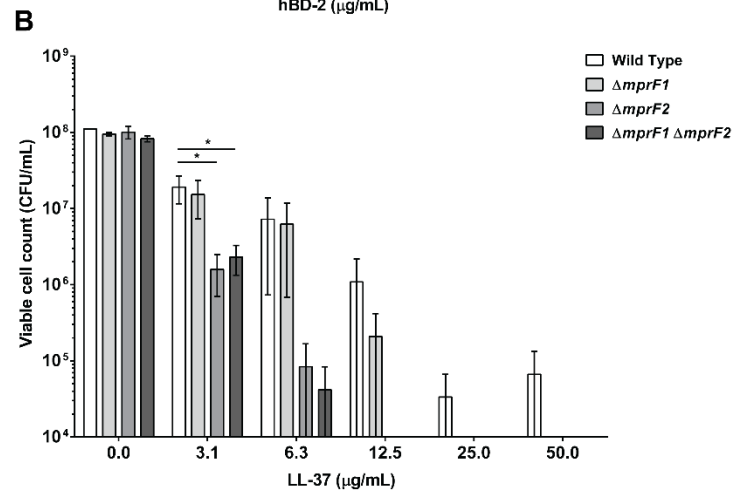

**C**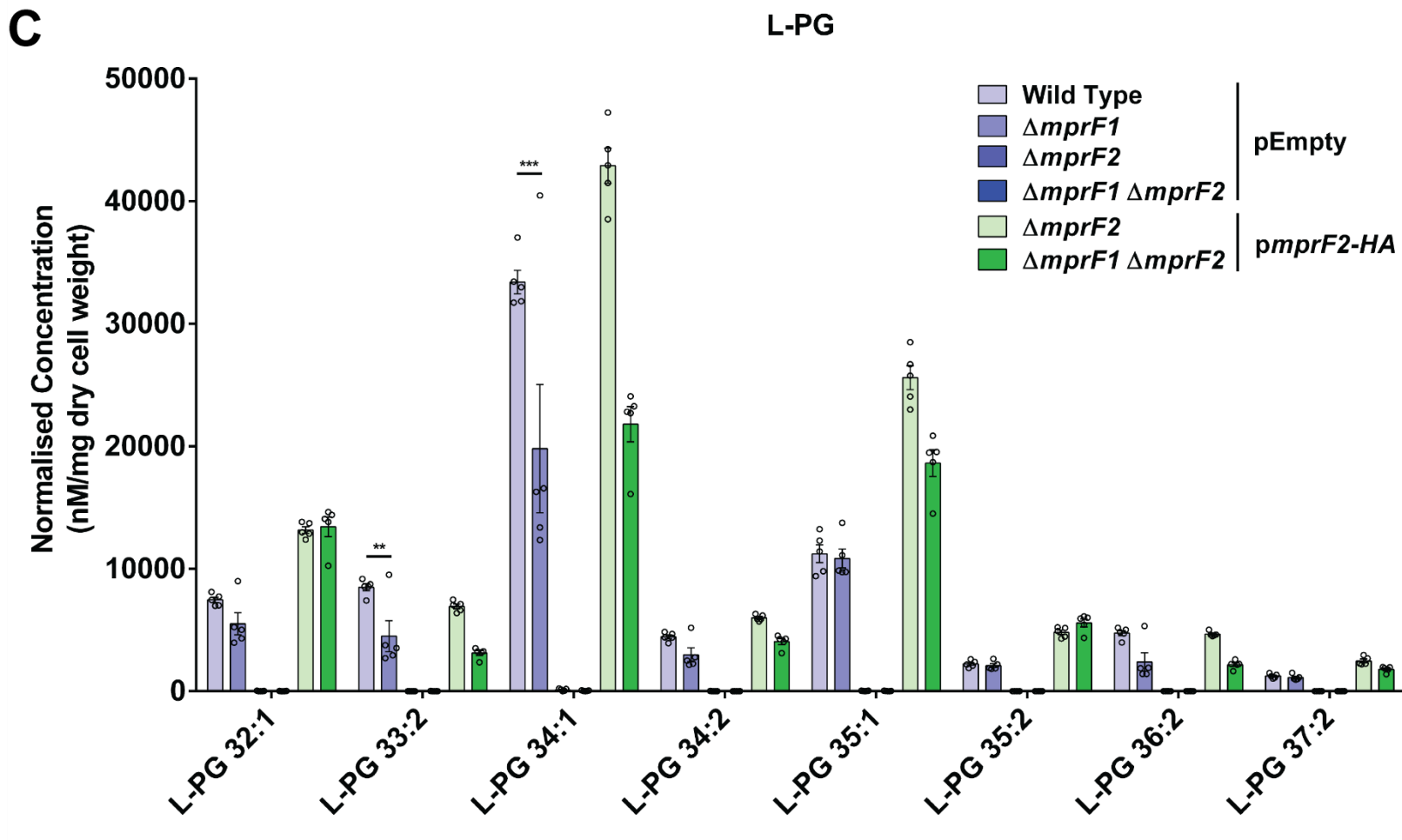

**D**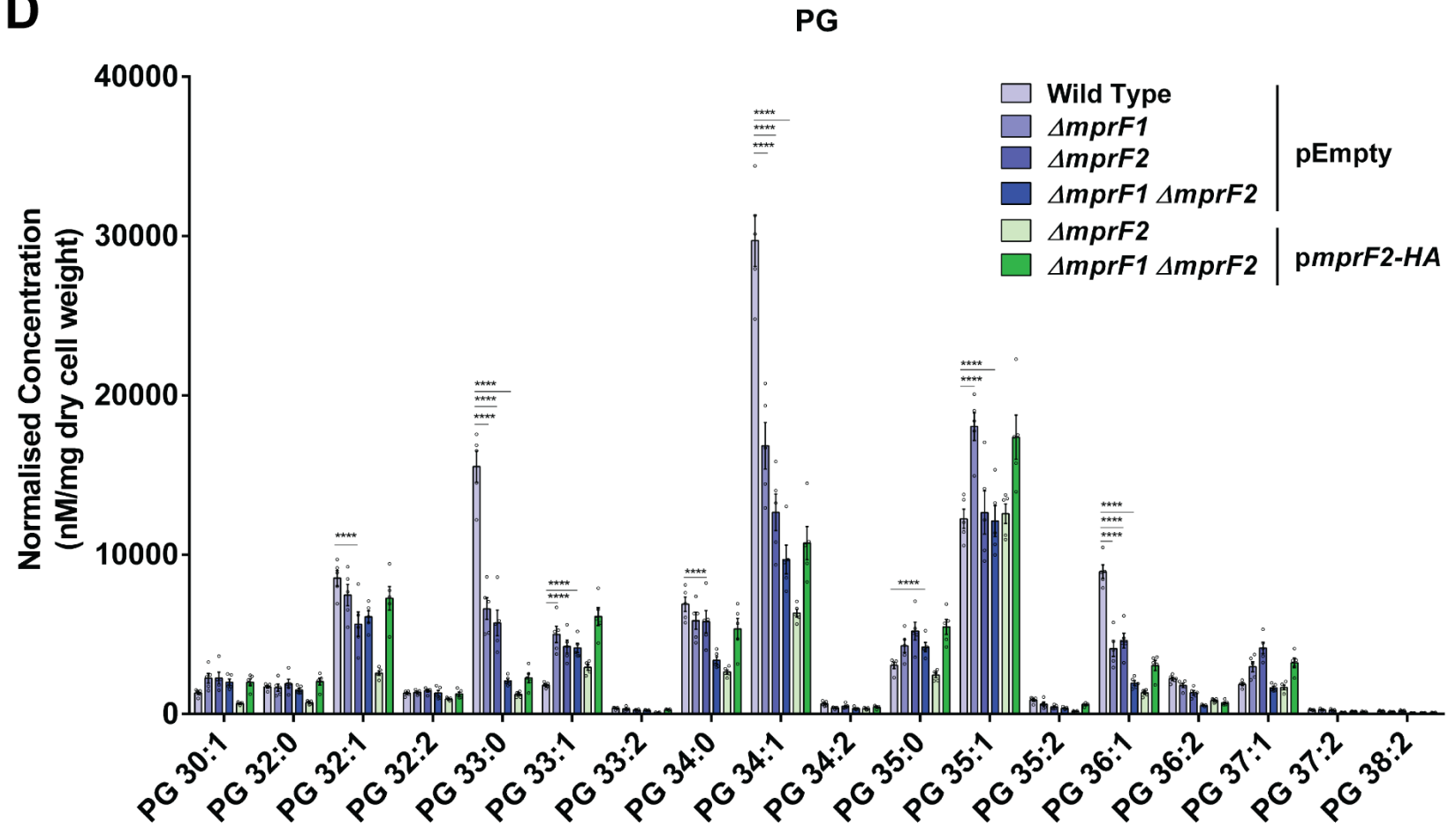

E

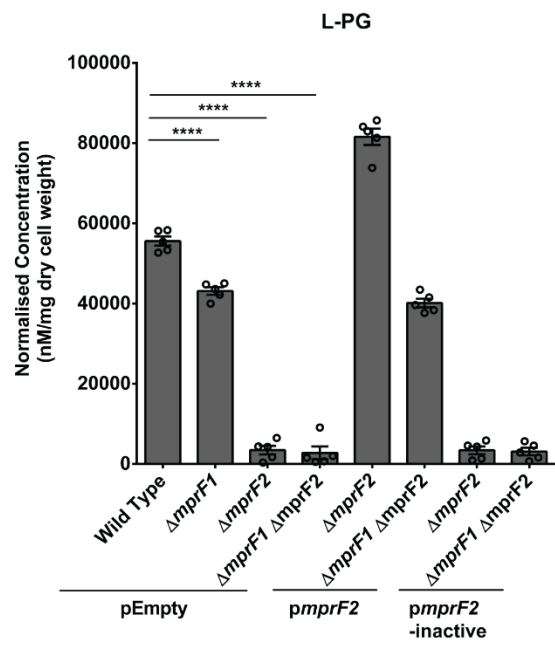

F

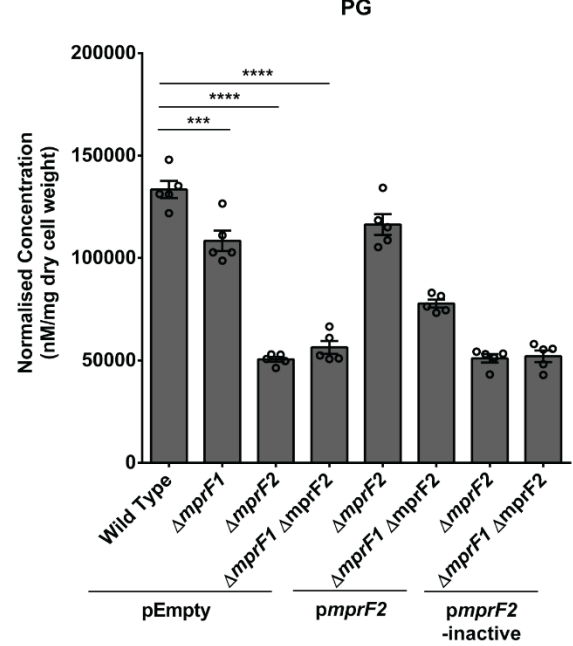

G

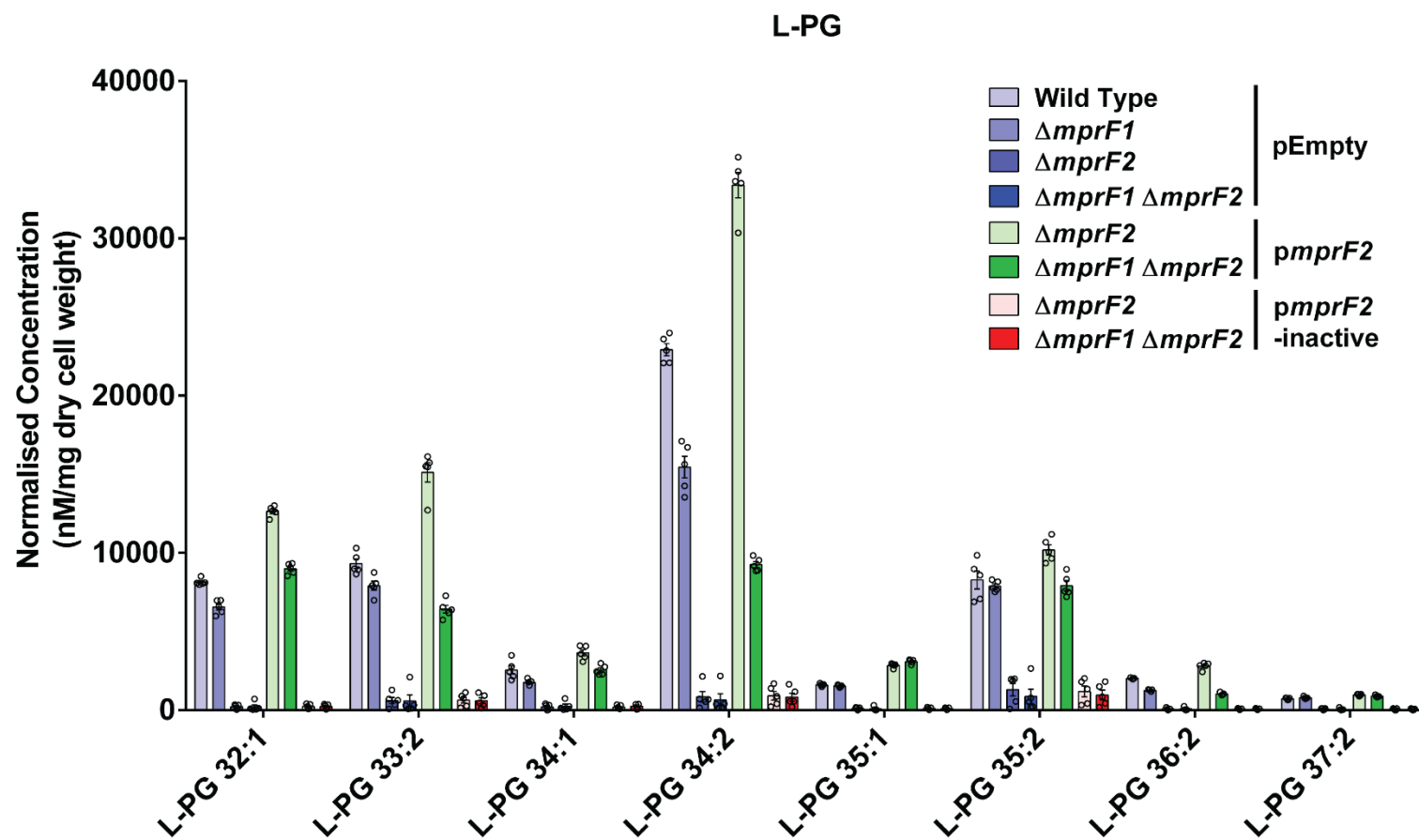

H

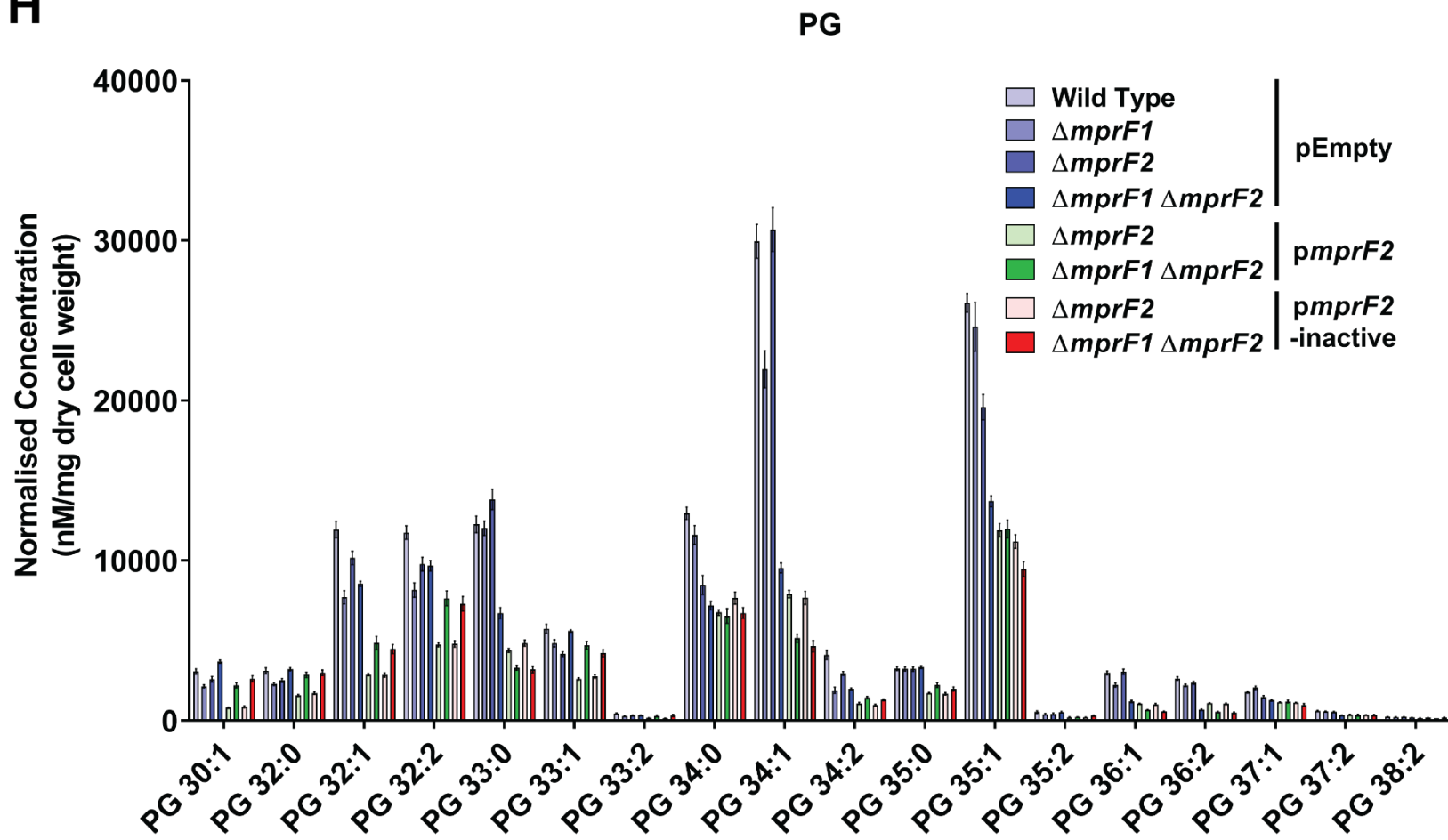

I

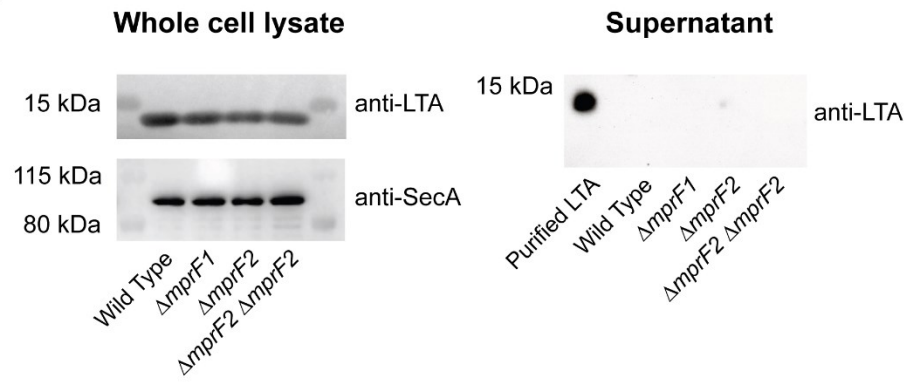

**J**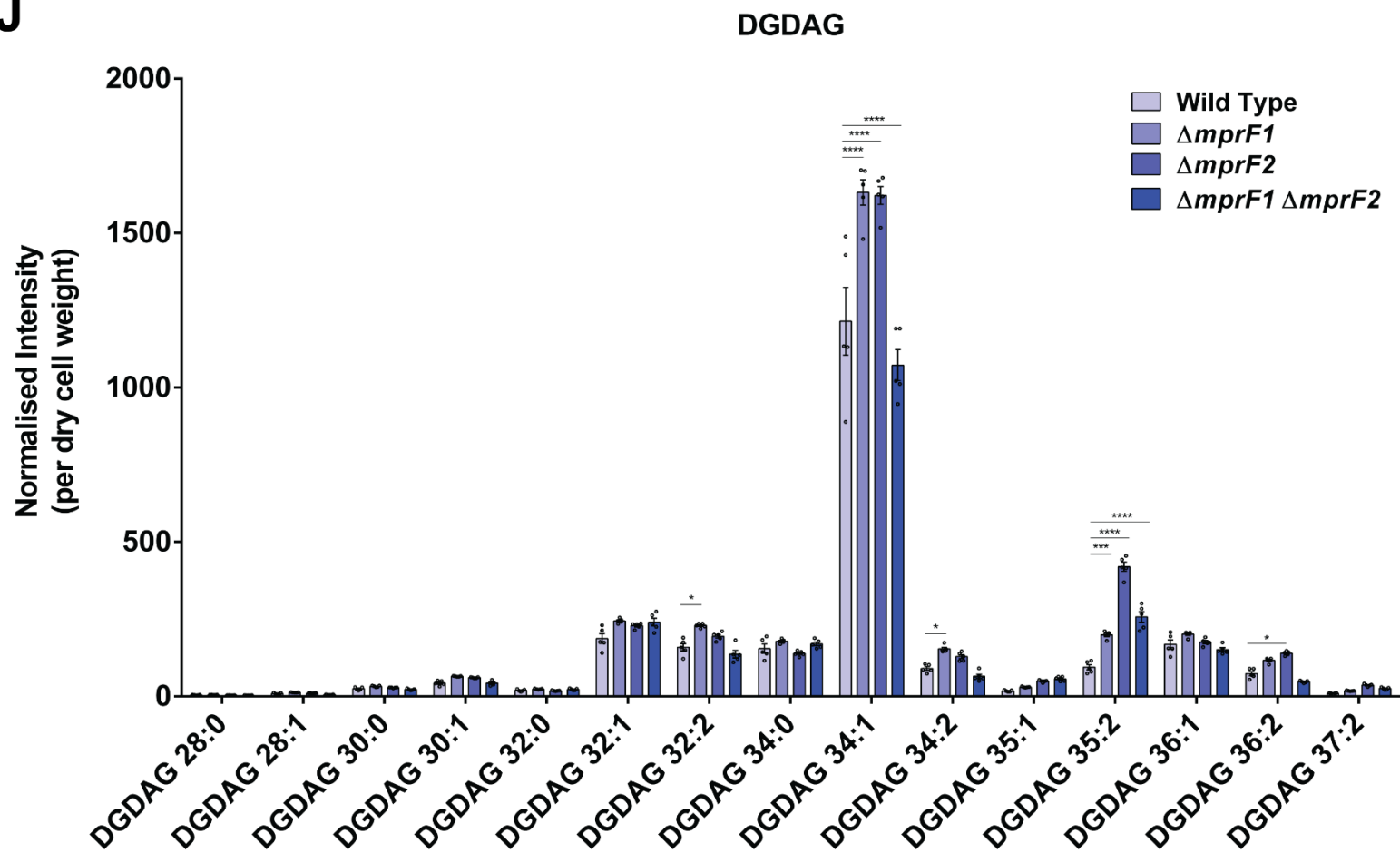

Supplement: FIG S1 [file mbio.03073-22-s0001.pdf]

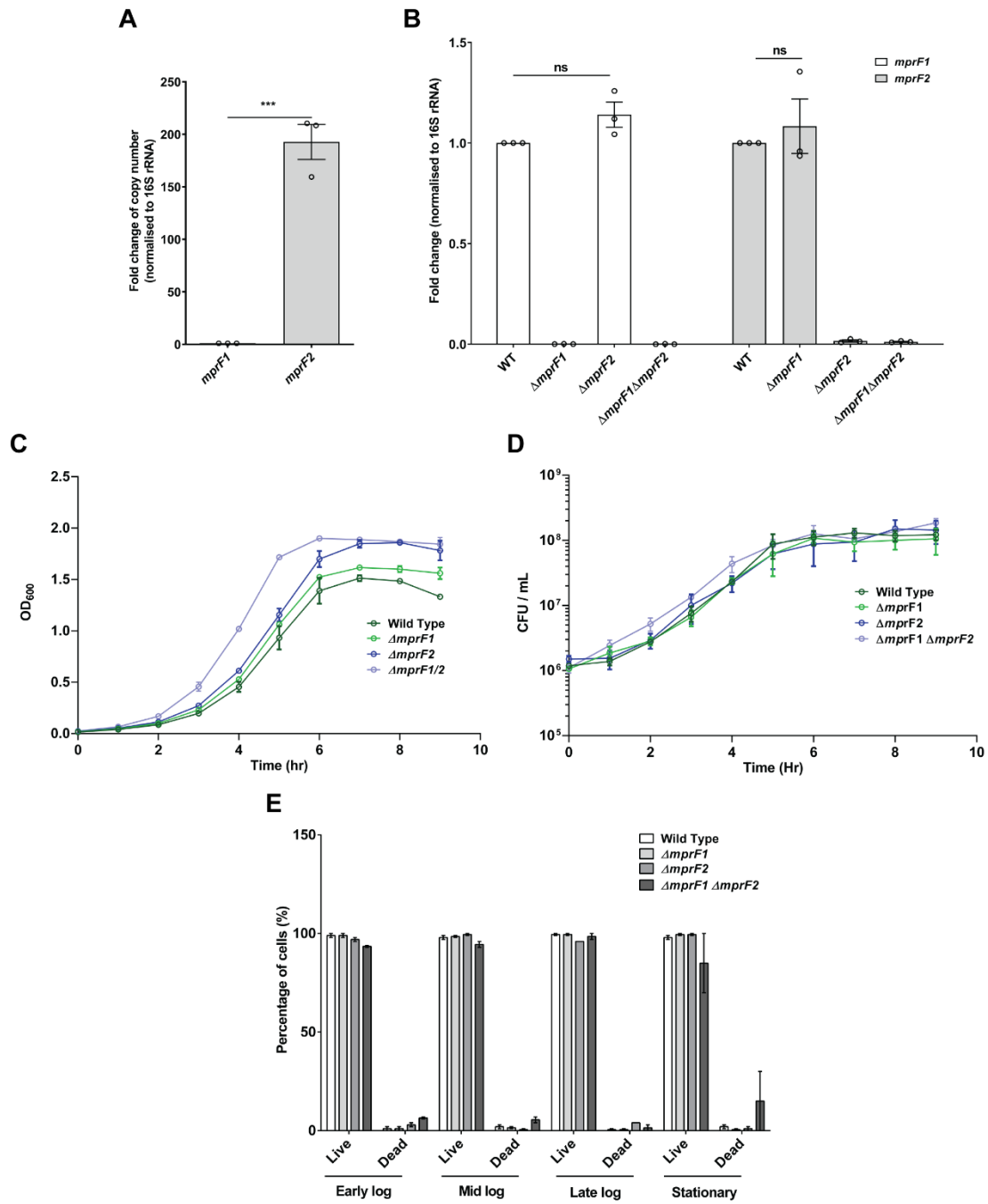

Supplement: FIG S2 [file mbio.03073-22-s0002.pdf]

**A**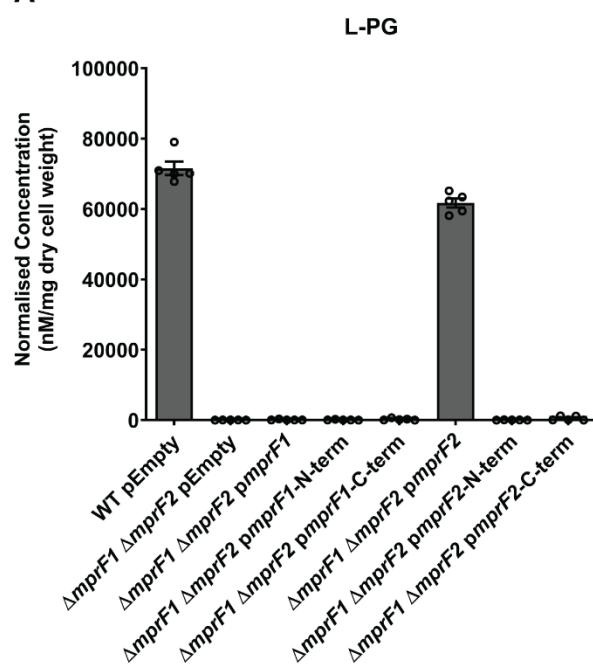**B**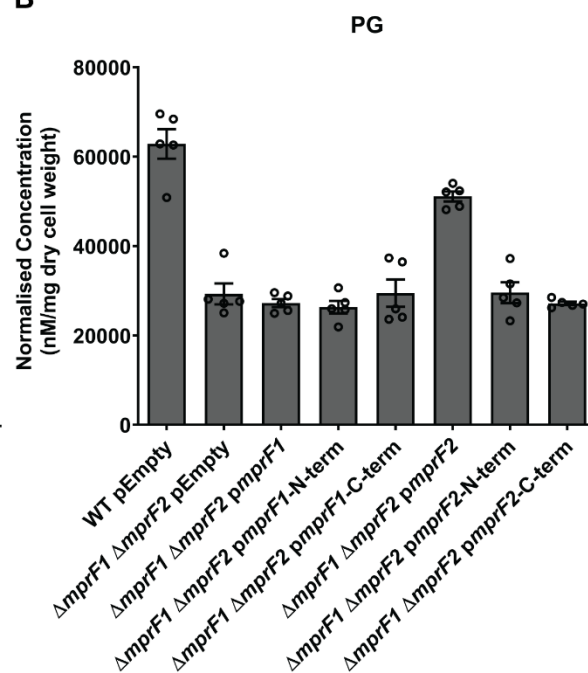

C

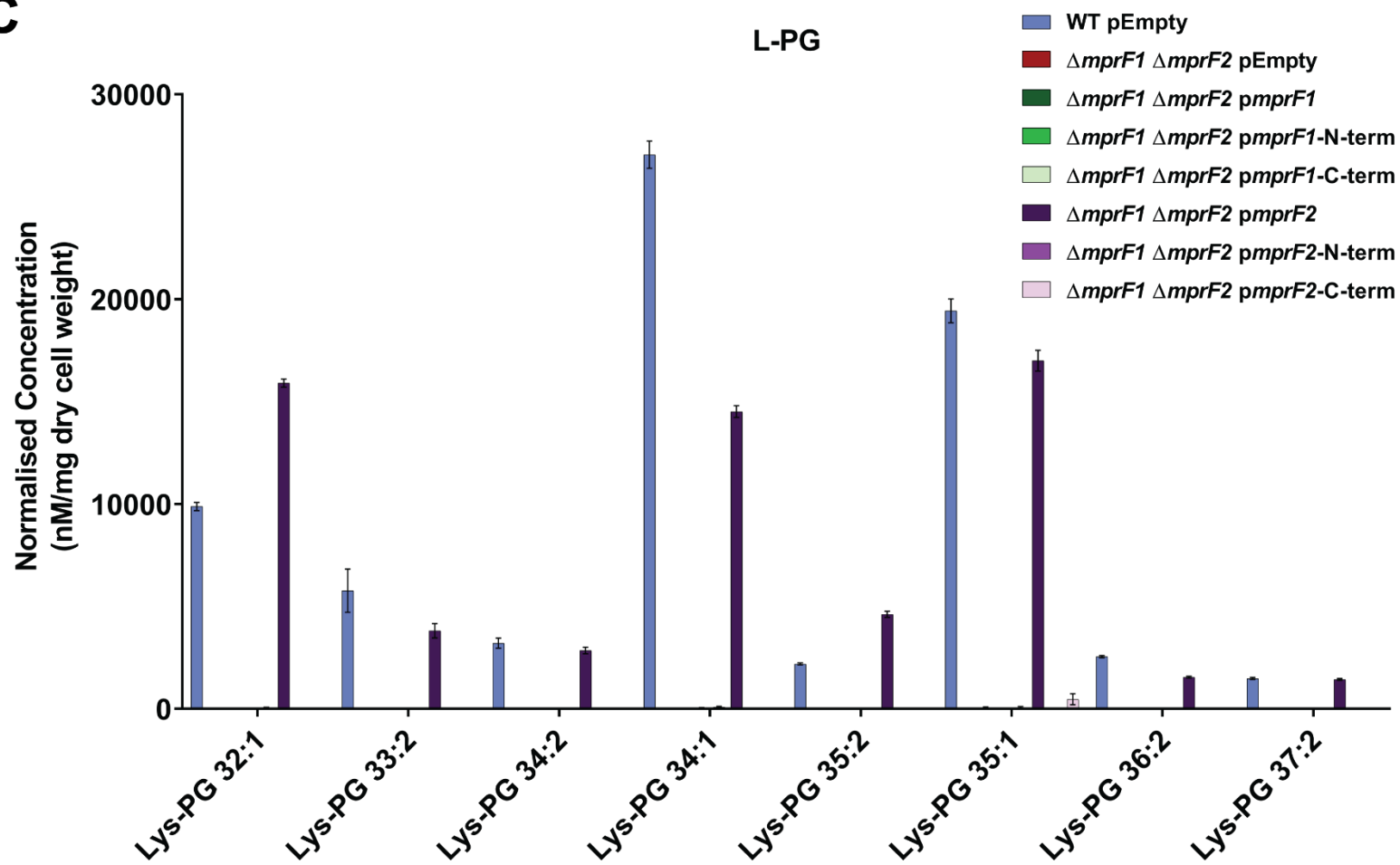

D

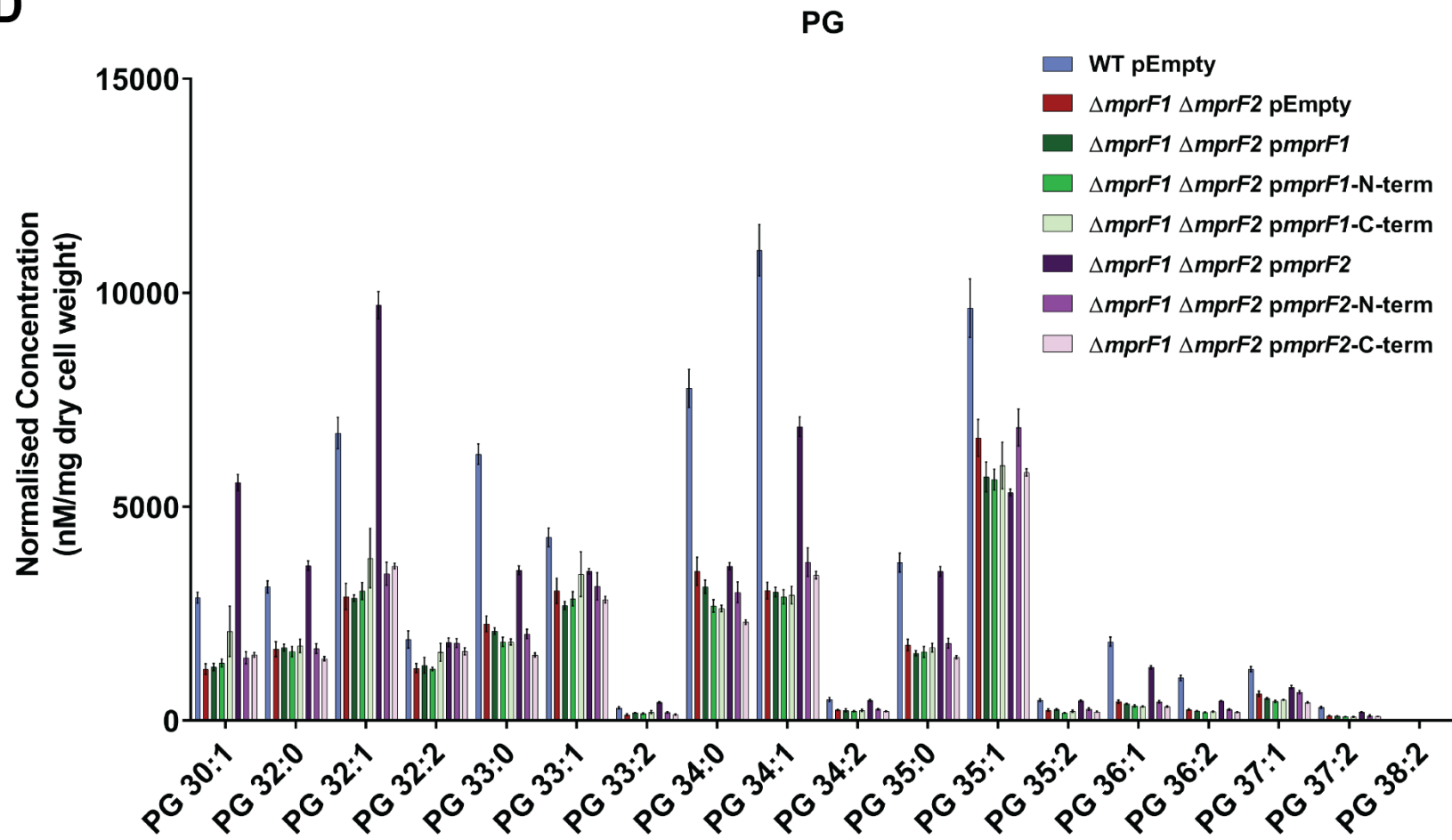

Supplement: FIG S3 [file mbio.03073-22-s0003.pdf]

**A**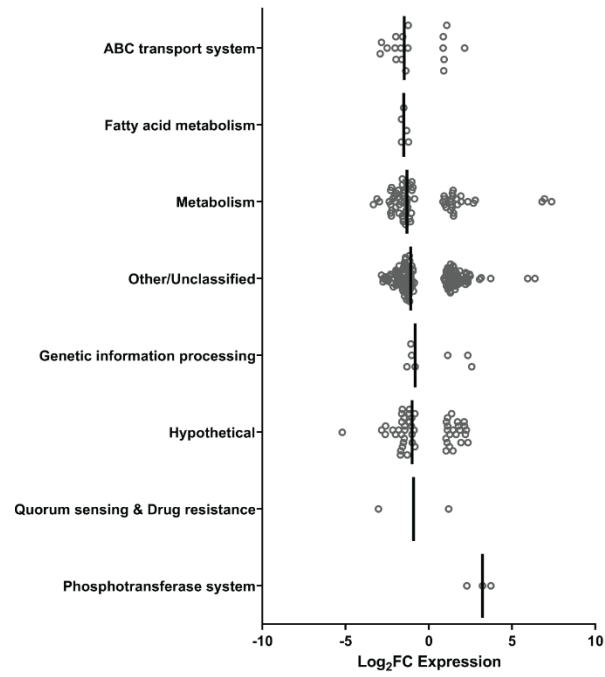**B**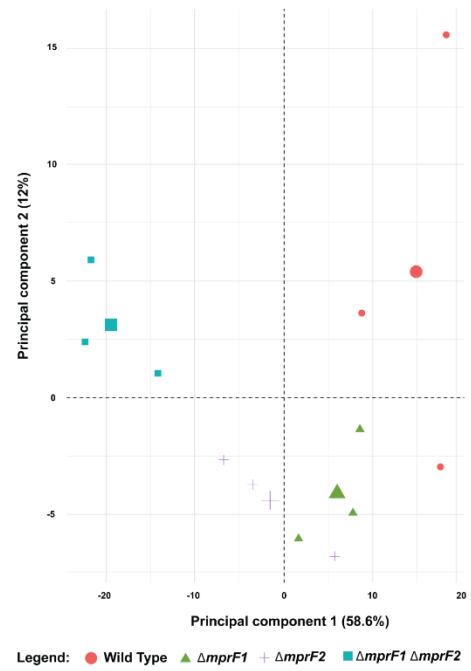**C** OG1RF genes differentially regulated in the *mprF* mutants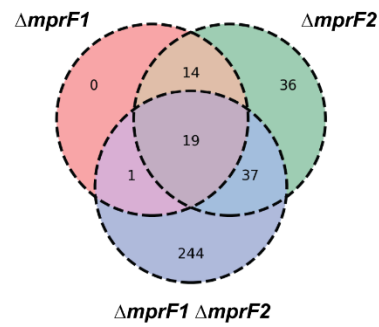**D**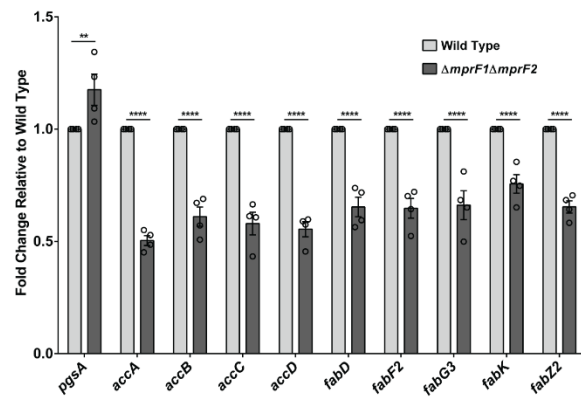

Supplement: FIG S5 [file mbio.03073-22-s0005.pdf]

**A**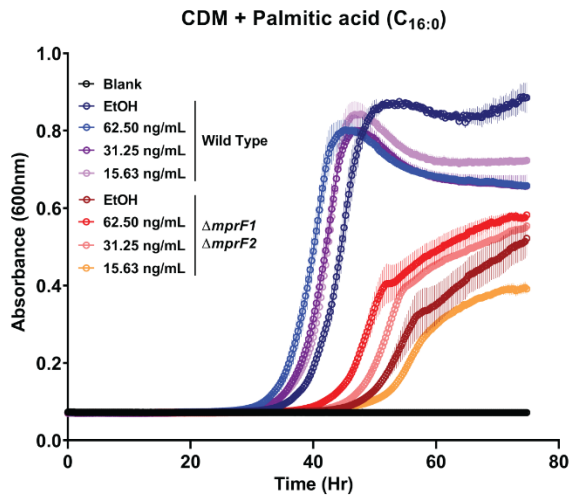**B**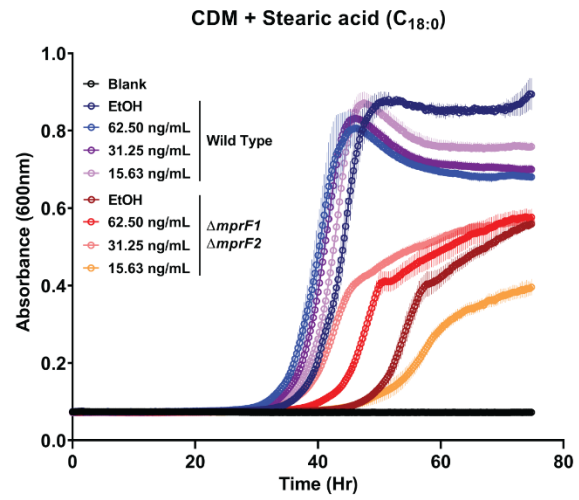**C**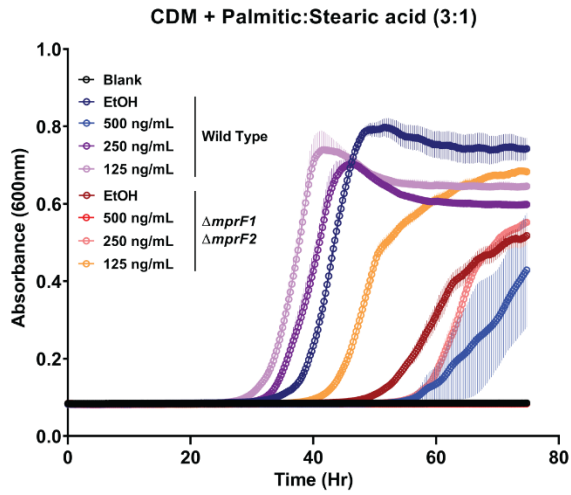**D**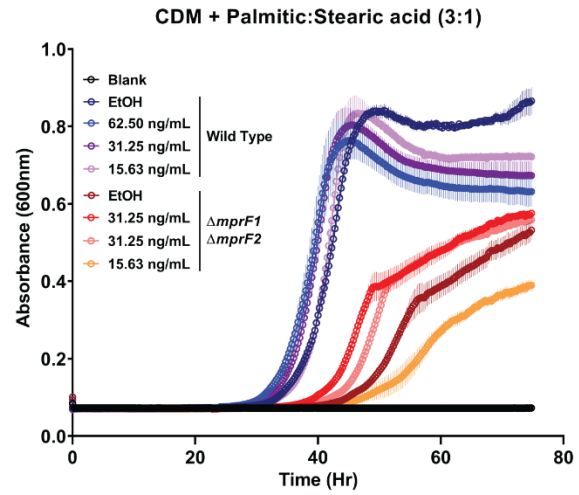

E

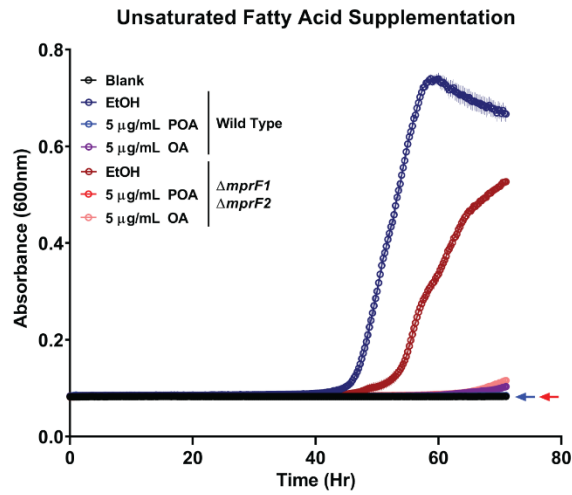

F

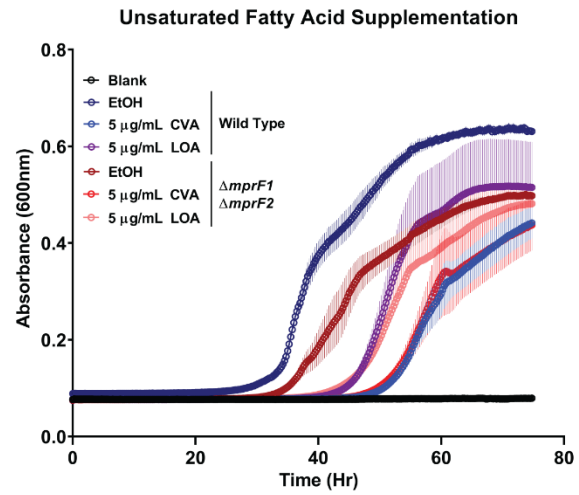

G

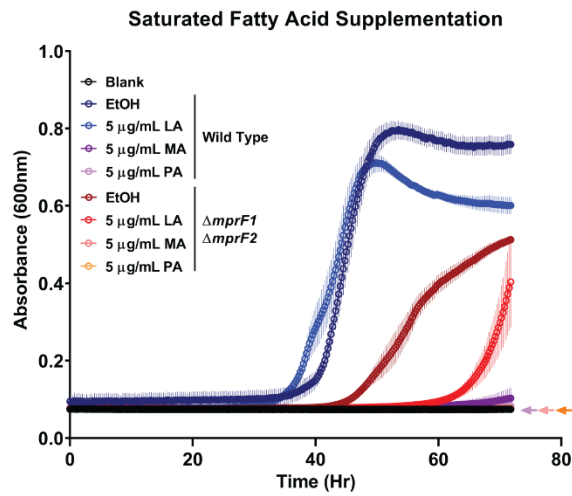

H

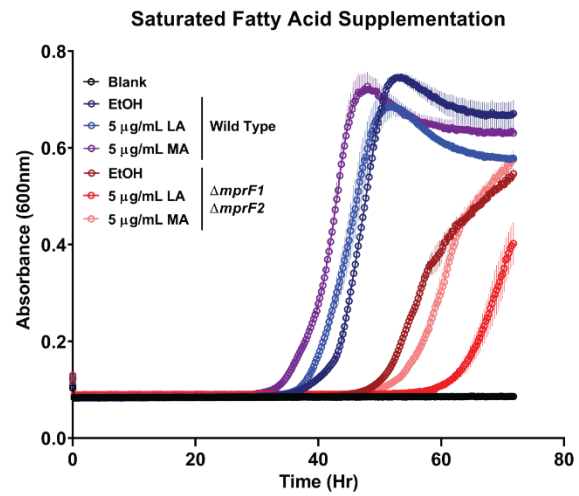

I

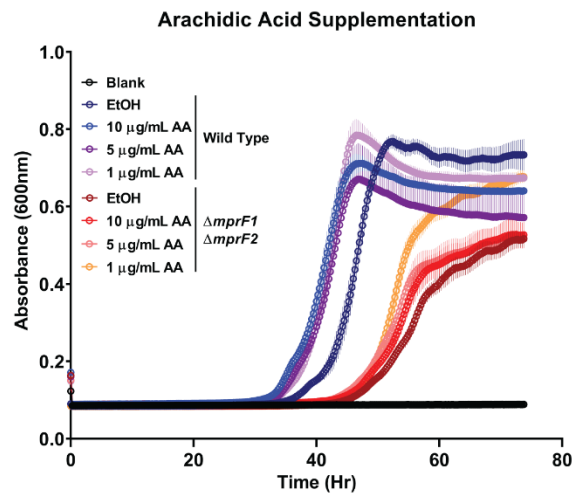

Supplement: FIG S6 [file mbio.03073-22-s0006.pdf]

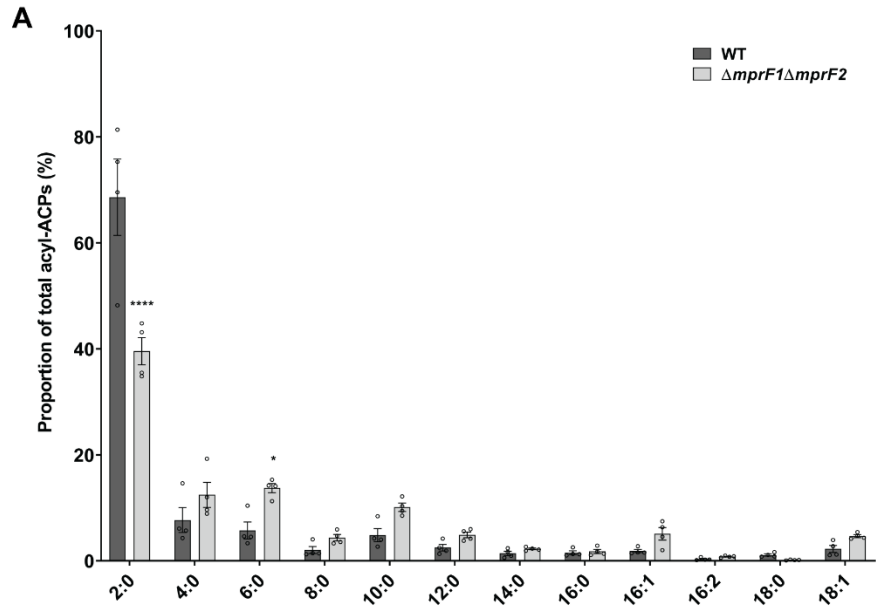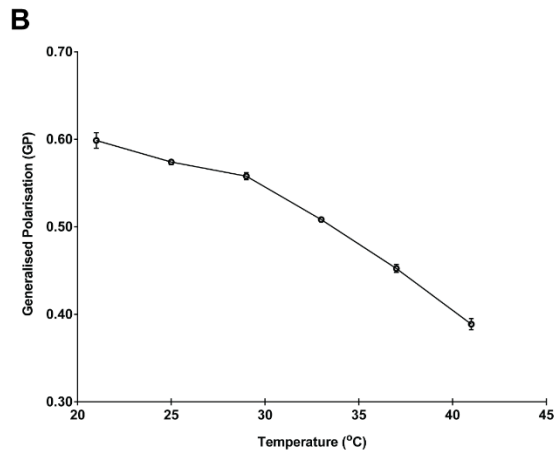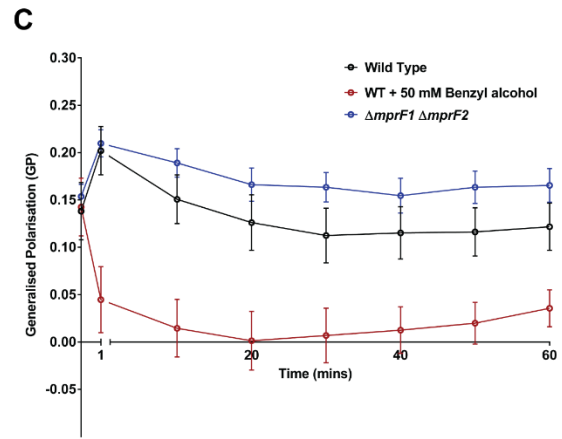

Supplement: FIG S7 [file mbio.03073-22-s0007.pdf]
