# Supplementary material for: Depleting Cationic Lipids Involved in Antimicrobial Resistance Drives Adaptive Lipid Remodeling in Enterococcus faecalis
Source: mBio. 2023 Jan 11;14(1):e03073-22. doi: 10.1128/mbio.03073-22 (PMC9973042; doi:10.1128/mbio.03073-22)

Chloroform: Methanol: Water (65:25:4) Solvent System

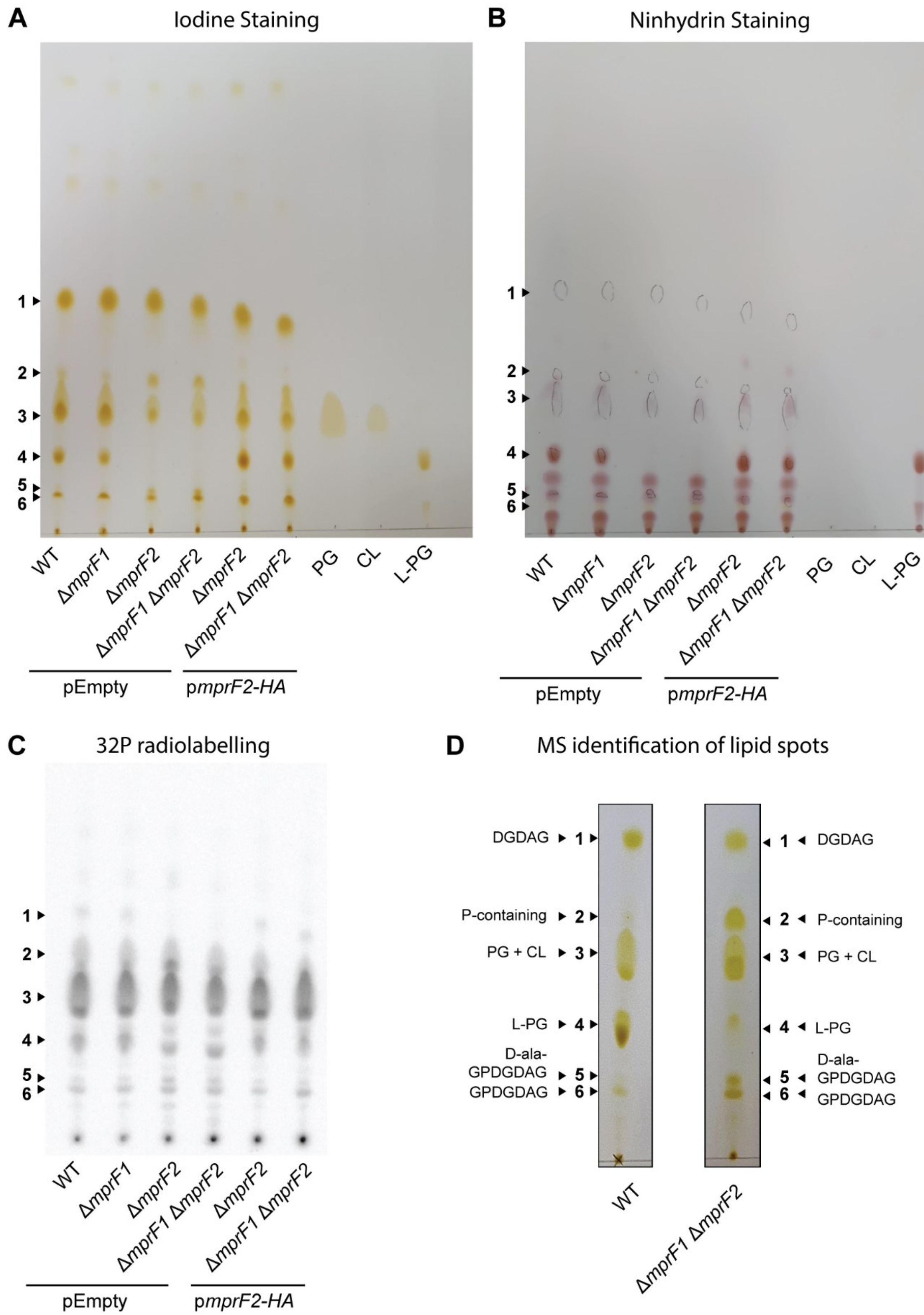

E

2 Dimension TLC

1st dimension = Chloroform: Methanol: Water (65:25:4)

2nd dimension = Chloroform: Hexane: Methanol: Acetic acid (50:30:10:5)

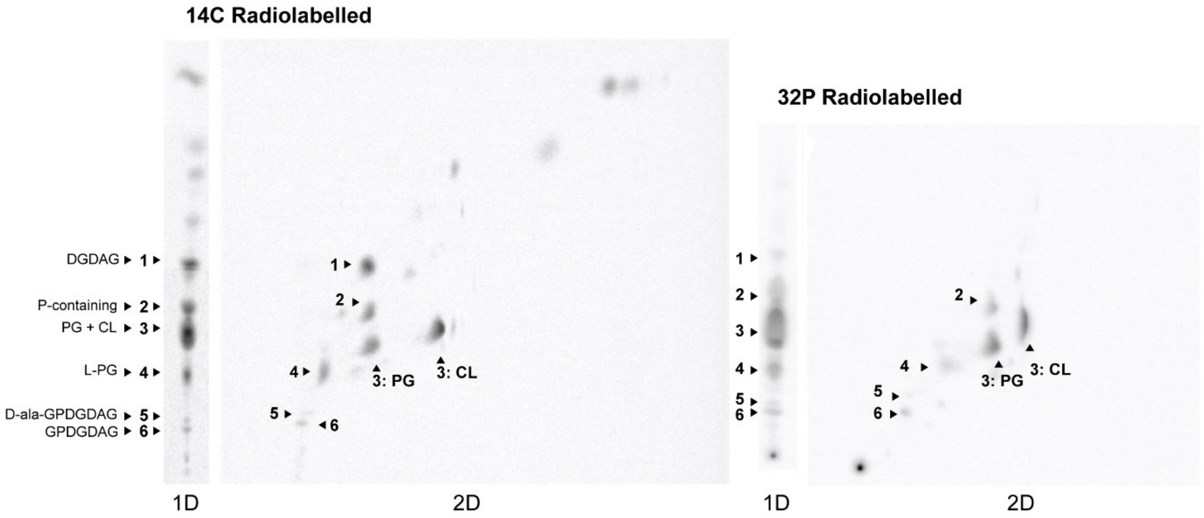

Supplement: FIG S4 [file mbio.03073-22-s0004.pdf]
